# Mating Leads to a Decline in the Diversity of Symbiotic Microbiomes and Promiscuity Increased Pathogen Abundance in a Moth

**DOI:** 10.3389/fmicb.2022.878856

**Published:** 2022-05-12

**Authors:** Luo-Yan Zhang, Hong Yu, Da-Ying Fu, Jin Xu, Song Yang, Hui Ye

**Affiliations:** ^1^Yunnan Academy of Biodiversity, Southwest Forestry University, Kunming, China; ^2^Key Laboratory for Forest Resources Conservation and Utilization in the Southwest Mountains of China, Ministry of Education, Southwest Forestry University, Kunming, China; ^3^School of Ecology and Environment, Yunnan University, Kunming, China

**Keywords:** *Spodoptera frugiperda*, reproductive microbiomes, mating, sexually transmitted microbes, pathogen, beneficial bacteria

## Abstract

Mating may promote microbial diversity through sexual transmission, while mating-induced immune responses may decrease it. Therefore, the study of mating-induced microbiomes changes under different mating systems is informative to unravel its biological relevance and evolutionary significance. Here, we studied the microbiomes in a community context within the abdomen of *Spodoptera frugiperda* females using 16S rDNA sequences by setting virgin females, and females mated once, twice, or thrice with the same or different males. Alpha and beta diversities revealed that mating significantly affected the composition of microbiomes in *S. frugiperda* females, wherein virgin females have the highest diversity, followed by one-time mated females and females mated with multiple males, while females mated repeatedly with the same male showed the lowest diversity. The low diversity in females mated repeatedly with the same male may be due to lower sexual transmission as only mated with one mate and higher immune response from repeated matings. Functional prediction by FAPROTAX and literature searching found 17 possible pathogens and 12 beneficial microbiomes. Multiple mating turned over the abundance of pathogens and beneficial microbes, for example, *Enterococcus* and *Lactobacillus* spp. (beneficial) showed higher abundance in virgin females while *Morganella* and *Serratia* spp. (pathogens) showed higher abundance in females mated with multiple males. These results suggest that mating causes a decline in the diversity of symbiotic microbiomes and promiscuity incurs a higher pathogen abundance in *S. frugiperda* females, which may be the result of sexual transmission of bacterial strains and immune responses targeting members of the microbiomes. To our knowledge, we demonstrate microbiomes changes in female insects under virgin and different mating regimes for the first time.

## Introduction

Animals live and evolve in a world dominated by microbes, from which they host a diversity of microbiomes on and in their bodies. It is now widely accepted that microbiomes can be an integral part of the host phenotype, which may influence the health, physiology, development, behavior, and reproduction of the host, and potentially influence the host genome and be a part of the host genome (Schoenmakers et al., 2019; Bellinvia et al., 2020a,b; Rowe et al., 2021). A wide range of studies so far have been performed to reveal the role of microbiomes on host skin, oral, and gut from perspectives of ecology, adaption, and evolution (Dillon and Dillon, 2004; Voirol et al., 2018; Rowe et al., 2021). However, reproductive microbiomes or reproduction-related microbiomes are relatively neglected and less known (Bellinvia et al., 2020a; Rowe et al., 2021). Reproductive microbiomes, including bacteria, fungi, viruses, protozoans, and unicellular algae, living in or on any structure, organ, fluid, or tissue of a host, typically make contact with the gametes or reproductive tract or organs of another individual through mating and spawning (Rowe et al., 2021).

Studies in mammals, particularly human, have demonstrated that a large number of sexually transmitted microbes have negative effects on the health and survival of hosts (Schoenmakers et al., 2019; Rowe et al., 2021). Fitness tests indicated that these microbes often can have significant effects (but not negative always) on the reproductive performance and success of males and females (Schoenmakers et al., 2019; Rowe et al., 2021). Evidence, in male mammals, increasingly recognized that some of these microbes may decrease sperm quality and fertility (Fraczek et al., 2013; Schoenmakers et al., 2019; Rowe et al., 2021) and, in female mammals, demonstrated that some microbes are associated with female health and reproduction, including sexually transmitted infections, preterm birth, infertility, and low fecundity (Lockhart et al., 1996; Ravel et al., 2011; Chen et al., 2017; Schoenmakers et al., 2019; Marconi et al., 2020). Studies in human also found that the typically dominated vaginal microbe, *Lactobacillus* spp., can be “healthy” vaginal microbiomes, which are oxygen-tolerant anaerobes that exhibit antimicrobial activity against a range of vaginal pathogens, probably by physically barring against pathogen adhesion, production of lactic acid, and stimulating host defense responses (Ravel et al., 2011; Chen et al., 2017; Tachedjian et al., 2017; Younes et al., 2018). Studies in this field are relatively limited in insects. A study in the bedbug showed that exposuring males to polymicrobial mixture (such as *Acinetobacter, Alcaligenes, Bacillus*, and *Staphylococcus*) significantly increased sperm mortality (up to 40%) (Otti et al., 2013). In some insects including some lepidopterans, *Wolbachia* infection may lead to increased survival and reproduction (Chen et al., 2012; Moriyama et al., 2015; Voirol et al., 2018), while some *Enterococcus* species, such as *E. faecalis*, may have a negative impact on the fecundity of fruit flies (Akami et al., 2019; Noman et al., 2021). Moreover, studies also found that reproductive-related microbiomes play a diversity of roles in the reproduction process of animals, including insects, which is related to sexual selection, sperm competition, genetic compatibility, sexual conflict, and speciation (Schoenmakers et al., 2019; Rowe et al., 2021). However, most studies examined bacteria effects with only one or a few types of bacteria (Rowe et al., 2021). One possible reason is that these microbes are large in quantity and species, and most of them are hard to cultivate or uncultivable (Bellinvia et al., 2020a). While the study of individual microbes is fundamental and informative, it is important to understand the microbiomes in a community context (Schoenmakers et al., 2019; Rowe et al., 2021).

Recent progress on high-throughput sequencing and bioinformatics has revolutionized our understanding of symbiotic microbiomes. In the field of reproductive microbiomes, a few studies have demonstrated the bacterial communities of the reproductive organs in insects using 16S rDNA sequencing and subsequent bioinformatic analysis (Wang et al., 2014; Otti et al., 2017; Bellinvia et al., 2020a,b). In the citrus fruit fly, *Bactrocera minax*, sequencing revealed that female ovary has the highest diversity in microbiomes, followed by male testis, and the bacterial diversity of reproductive organs is higher than that of the gut (Wang et al., 2014). Recent studies in the common bedbug, *Cimex lectularius*, showed that genital microbiomes varied between populations and the sexes and mating changes the genital microbiomes, suggesting that bacteria are sexually transmitted and these bacteria might play an important role in shaping the evolution of reproductive traits (Bellinvia et al., 2020a,b). Future studies based on these new findings and technologies are likely to provide deeper insights in this field by investigating the transmission dynamics between males and females.

Mating can cause major changes in female physiology and behavior. Studies have shown that mating has a positive effect on female reproductive activities, which may negatively affect female maintenance (such as immunity responses) and survival due to trade-offs on resource utilization by females, or male suppression of female immunity to promote sperm storage and egg fertilization in females (reviewed in Schwenke et al., 2016; Wigby et al., 2019). However, studies have also shown that mating may upregulate the female's immune response due to the transfer of foreign materials and mating caused infections (Delbare et al., 2017; Okada et al., 2017; Oku et al., 2019; Ahmed-Braimah et al., 2021). These different discoveries may stem from different mating systems. Females of polygamous species should have higher postmating immunity if sexually transmitted infection is the major factor driving female postmating costs (Oku et al., 2019). Therefore, it is imperative and informative to study reproductive microbiomes as a whole in a community context in different mating systems (such as polygamy or polyandry) and under different mating conditions (such as repeated mating or multiple mating). Male mating with more than one female is known as polygamy, while female mating with more than one male is known as polyandry. Multiple mating (mating with more than one mate) and repeated mating (mating repeatedly with the same mate) are common in both sexes in insects, birds, and mammals (Andersson, 1994).

As mentioned above, studies have studied the microbiomes in the reproductive organs and guts in mated and virgin insects (Wang et al., 2014; Bellinvia et al., 2020a,b). However, in addition to reproductive organs and guts, symbiotic microbiomes also exist in other tissues or organs, either intracellularly or extracellularly, such as in the fat body and hemolymph (Szklarzewicz and Michalik, 2017). All these symbiotic microbiomes are under the surveillance of insect immune system and thus are likely to be affected by the mating-induced defense responses (Whitlow, 2004; Okada et al., 2017; Oku et al., 2019). Therefore, in this study, we studied the symbiotic microbiomes in the abdomen of *Spodoptera frugiperda* females by evaluating microbiomes as a whole in a community context. To test the above signified hypotheses, we used virgin females as controls, treat other females to mate once, twice, or thrice with the same (repeated mating) or different males (multiple mating), and then measured the exact changes on microbiomes in female abdomen using 16S rDNA sequencing. The evolutionary significance of reproductive microbiomes and interactions between host physiology and microbial communities were further explored based on these data.

The fall armyworm, *Spodoptera frugiperda* (Lepidoptera: Noctuidae), is native to tropical and subtropical regions from Argentina to the United States in the Western Hemisphere (Kumar et al., 2009). *S. frugiperda* is a long-distance migratory pest, but there was no report on its distribution outside the Americas before 2015. This pest was first discovered in Africa in 2016 (Abrahams et al., 2017) and then invaded India and some other areas in Asia in 2018 (FAO, 2019; CABI, 2020). Soon thereafter, this pest was discovered in Yunnan Province of China at the end of 2018 and then quickly spread northward to the vast areas of China (Guo et al., 2019; Xu et al., 2019; Zhang et al., 2019). This pest mainly damages corn and wheat in China, with the annual potential loss being $17,286–52,143 m for corn (Qin et al., 2020) and $15,571–90,143 m for wheat (Xu et al., 2020), respectively. Symbiotic microbiomes play imperative roles in the survival and reproduction of the host, in addition to the interactions of insect pests, host plants, and natural enemies (Ferrari and Vavre, 2011; Perilla-Henao and Casteel, 2016; Beck and Vannette, 2017). Therefore, modifying symbiotic microbiomes is a potential management strategy for the control of agricultural insect pests (Perilla-Henao and Casteel, 2016; Beck and Vannette, 2017).

## Materials and Methods

### Insect Rearing and Sampling

*Spodoptera frugiperda* larvae were collected in July 2019 on corn plants in the field near Zhanyi town in Yunnan Province, China. The insects were reared on the artificial diet (Li et al., 2006) under 28 ± 1°C and 60–80% relative humidity with 14:10-h light:dark photoperiod, in the IPM Laboratory of Southwest Forestry University (Kunming, China). This insect has 11 generations in the laboratory before this study that was performed in January 2021. All insects used for sequencing are reared using the same method under the same condition as above, which minimized the random effects from the insect populations and environment.

**Figure 1:**
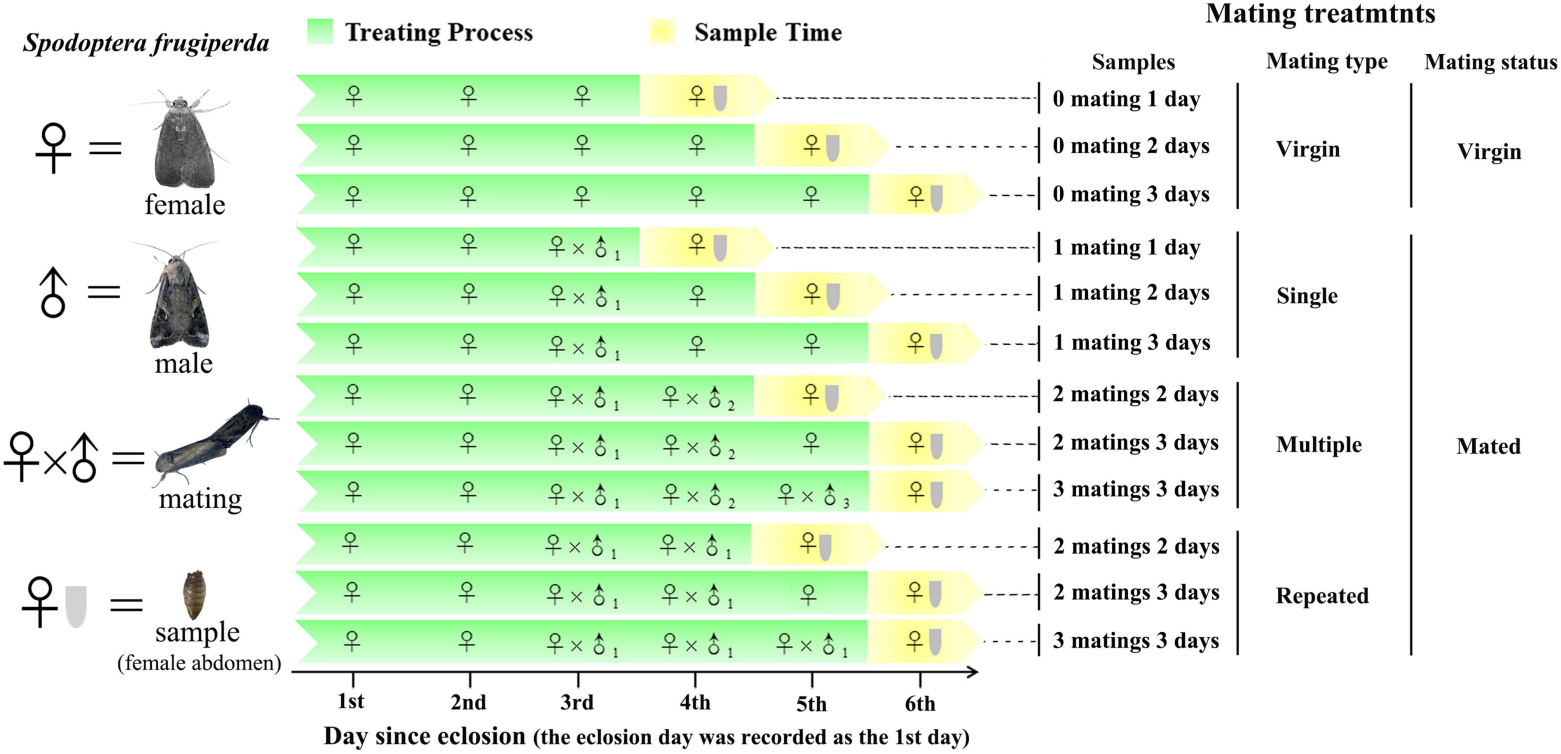
Experimental design and sampling methods. Single refer to one-time mated females, in which 1 mating 1 day (*n* = 3), 1 mating 2 days (*n* = 3), and 1 mating 3 days (*n* = 3) refer to one-time mated females sampled at 1, 2, and 3 d postmating (since the first mating), respectively. Multiple refer to females mated with multiple males (one male one mating), in which 2 matings 2 days (*n* = 3), 2 matings 3 days (*n* = 3), and 3 matings 3 days (*n* = 3) refer to twice and thrice mated females sampled at 2 and 3 d postmating (since the first mating), respectively. Repeated refer to females mated repeatedly with the same male, in which 2 matings 2 days (*n* = 3), 2 matings 3 days (*n* = 3), and 3 matings 3 days (*n* = 3) refer to twice and thrice mated females sampled at 2 and 3 d postmating (since the first mating), respectively. Virgin refer to unmated females, in which 0 mating 1 day (*n* = 3), 0 mating 2 days, and 0 mating 3 days (*n* = 3) refer to virgin females sampled at 1, 2, and 3 d postmating (relative to the above mating treatments), respectively.

To ensure virginity, male and female pupae were sexed based on morphological characteristics (Dong et al., 2019) and then were caged separately. Adult eclosion was recorded daily, and the eclosion day was recorded as the 1st day since eclosion (Figure 1). Females were allowed to mate once, twice, and thrice with the same or different males within the 4th to 6th day since eclosion. A female mated more than once with the same mate was recognized as repeated mating, while a female mated more than once with different mates was recognized as multiple mating. Virgin females were used as controls. Three replicates were used for each treatment, and six females were used for each replicate. Before abdomen sampling, the whole body of females was rinsed twice with sterile water and was surface-sterilized in 75% ethanol for 90 s and then rinsed twice again using sterile water. The abdomens were then cut from the sterilized females using sterile scissors and were stored at – 80°C until use.

### DNA Extraction and 16S rDNA Sequencing

Total genome DNA was extracted from samples using CTAB method (Doyle and Doyle, 1987). The 16S rDNA regions were amplified via PCR with the V3–V4 primers (515F: 5′-GTGYCAGCMGCCGCGGTAA-3′; 806R:5′-GGACTACNNGGGTATCTAAT-3′) and with Phusion High-Fidelity PCR Master Mix (New England Biolabs, USA). The PCR products were analyzed using 2% agarose gel electrophoresis and were purified using a GeneJET Gel Extraction Kit (Thermo Scientific, USA). Sequencing libraries were generated using a TruSeq DNA PCR-Free Library Preparation Kit (Illumina, USA), according to the manufacturer's recommendations. Library quality was assessed on a Qubit Fluorometer (Thermo Scientific, USA) and an Agilent Bioanalyzer 2100 system (Agilent Technologies, USA). Finally, the library was sequenced on an Illumina NovaSeq PE250 platform (Novogene Bioinformatics Technology Co., Ltd., Beijing, China). Double-distilled water (ddH_2_O) was used as a negative control for DNA extraction and PCR amplification. Electrophoresis did not show any band or smudge in the negative control. No negative controls were used for library construction and sequencing, and no positive controls were used for DNA extraction or sequencing. The obtained raw data were deposited into the NCBI Sequence Read Archive (SRA) database (Accession No.: PRJNA742857).

### Data Analysis

Clean reads were obtained by removing chimera sequences and low-quality reads from the raw reads. Uparse software (Uparse v7.0.1001, http://drive5.com/uparse/) (Edgar, 2013) was used for subsequent sequence analysis. The sequences with ≥ 97% similarity were assigned to the same OTUs. The representative sequence for each OTU was screened for further annotation using Silva 138 (https://www.arb-silva.de/) (Wang et al., 2007) based on the Mothur algorithm.

The QIIME (Version 1.7.0) (Caporaso et al., 2010) was used for the analysis of alpha diversity to reveal the complexity of species within samples. The differences of Shannon diversity indices between different mating treatments (Figure 1) were analyzed using an ANOVA followed by LSD tests with Bonferroni correction for multiple comparisons.

Beta diversity was applied to assess the differences of microbial community between treatments. The significance of differences between treatments was tested using non-parametric multivariate analysis of variance (NPMANOVA) based on Bray–Curtis metrics and then visualized accordingly using the principal coordinates analysis (PCoA) based on Bray–Curtis metrics. NPMANOVA was performed using R (Version 4.0.3) (Team, 2020) with vegan (Dixon, 2003) and phyloseq (Mcmurdie and Holmes, 2013) packages.

The linear discriminant analysis (LDA) of effect size (http://huttenhower.sph.harvard.edu/galaxy/) was used to determine OTUs that discriminate among the populations with an LDA score is more than 3.0. A cladogram was also constructed to show the LDA results.

FAPROTAX (Louca et al., 2016) was used to predict the potential functional annotation of taxa in different size fractions, which predicts functions of uncultured prokaryotes from the known functions of cultured bacterial genera.

## Results

### Sequencing and Quality Control

By 16S rDNA gene sequencing using the Illumina NovaSeq platform, ~70,000 clean reads were obtained from each of the 36 sequenced libraries, with the average length being 424–429 bp (Supplementary Table 1). The percentages of Q20 and Q30 of all samples' clean reads ranged from 96.60 to 97.53% and from 90.62 to 92.56%, respectively. These sequences were clustered into 2005 OTUs (Supplementary Table 2). The rarefaction analysis showed a saturating number of OTUs (Supplementary Figure 1), which indicates an adequate sequencing output for all samples.

### Diversity Indices of Bacterial OTUs

**Figure 2:**
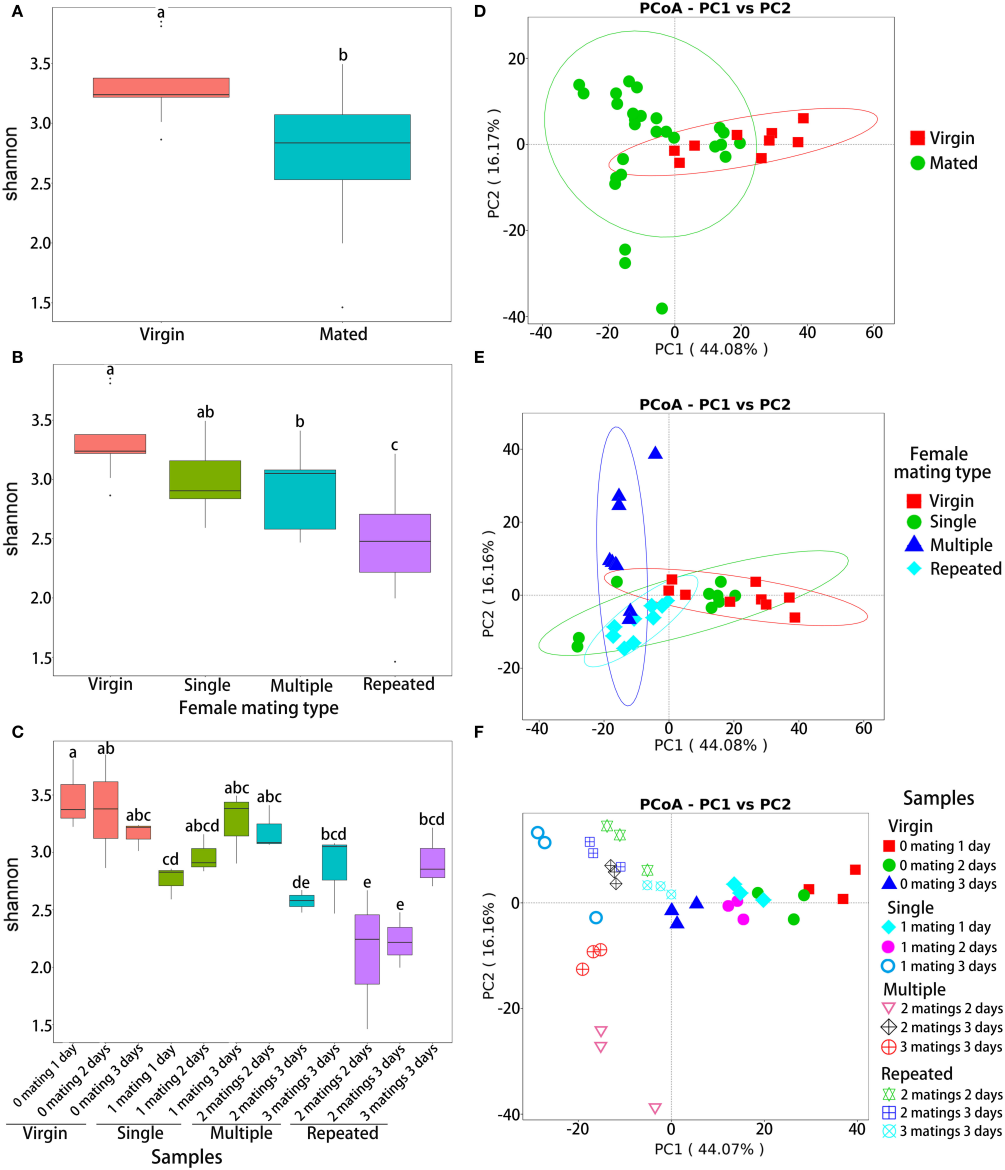
Diversity indices of bacterial OTUs in *S. frugiperda* females with different mating treatments. Shannon diversity indices of virgin and mated females **(A)**, females with different mating types **(B)**, and all samples **(C)**. PCoA ordination based on Bray–Curtis distances of virgin and mated females **(D)**, females with different mating types **(E)**, and all samples **(F)**. In each of the subgraphs of (a) to (c), bars with different letters are significantly different (*P* < 0.05).

Analysis of variance based on Shannon diversity metrics indicated that the OTU abundance was significantly different between virgin and mated females (ANOVA: *F*_1,34_ = 11.8, *P* = 0.002; Figure 2A), among females of different mating types (ANOVA: *F*_4,32_ = 8.808, *P* < 0.0001; Figure 2B) and among different samples (ANOVA: *F*_11,24_ = 5.061, *P* < 0.0001; Figure 2C). Within different mating types (Figure 2B), *post-hoc* pairwise comparison by LSD test (Supplementary Table 3) showed that virgin females (Virgin) have the highest abundance (*P* < 0.05), followed by one-time mated females (Single) and females mated with multiple males (Multiple) (*P* < 0.05), while females mated repeatedly with the same male (Repeated) showed the lowest abundance (*P* < 0.05). Within different samples (Figure 2C), *post-hoc* LSD test (Supplementary Table 4) also showed a similar decline trend on abundance, that is, samples of Virgin showed the highest abundance, followed by samples of Single, and then samples of Multiple, while samples from Repeated showed the lowest diversity (*P* < 0.05).

Beta-diversity analysis based on Bray–Curtis distance (illustrated by PCoA) also showed significant variances in the composition of OTUs between virgin and mated females (NPMANOVA: *F*_1,34_ = 3.00, *R*^2^= 0.081, *P* = 0.019; Figure 2D), among females of different mating types (NPMANOVA: *F*_3,32_ = 6.506, *R*^2^= 0.379, *P* < 0.001; Figure 2E) and among different samples (NPMANOVA: *F*_11,24_ = 14.036, *R*^2^= 0.865, *P* < 0.001; Figure 2F).

### Taxonomy Assignment

**Figure 3:**
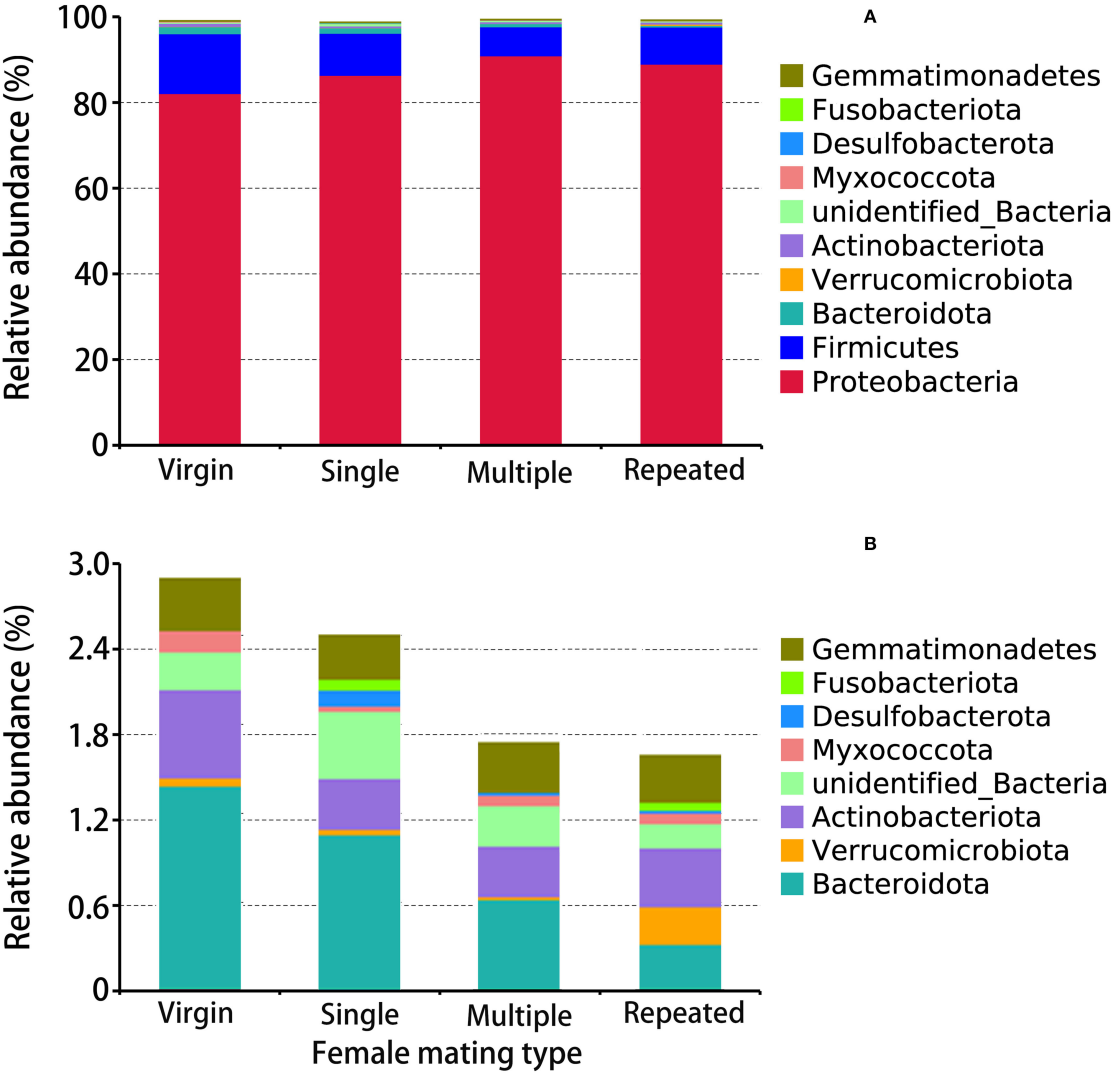
Taxonomy assignment of bacterial OTUs at the phylum level in *S. frugiperda* females of different mating types. **(A)** The abundance pattern of the top 10 phyla. **(B)** A clearer display of the relative abundance of the 3rd to the 10th phylum.

**Figure 4:**
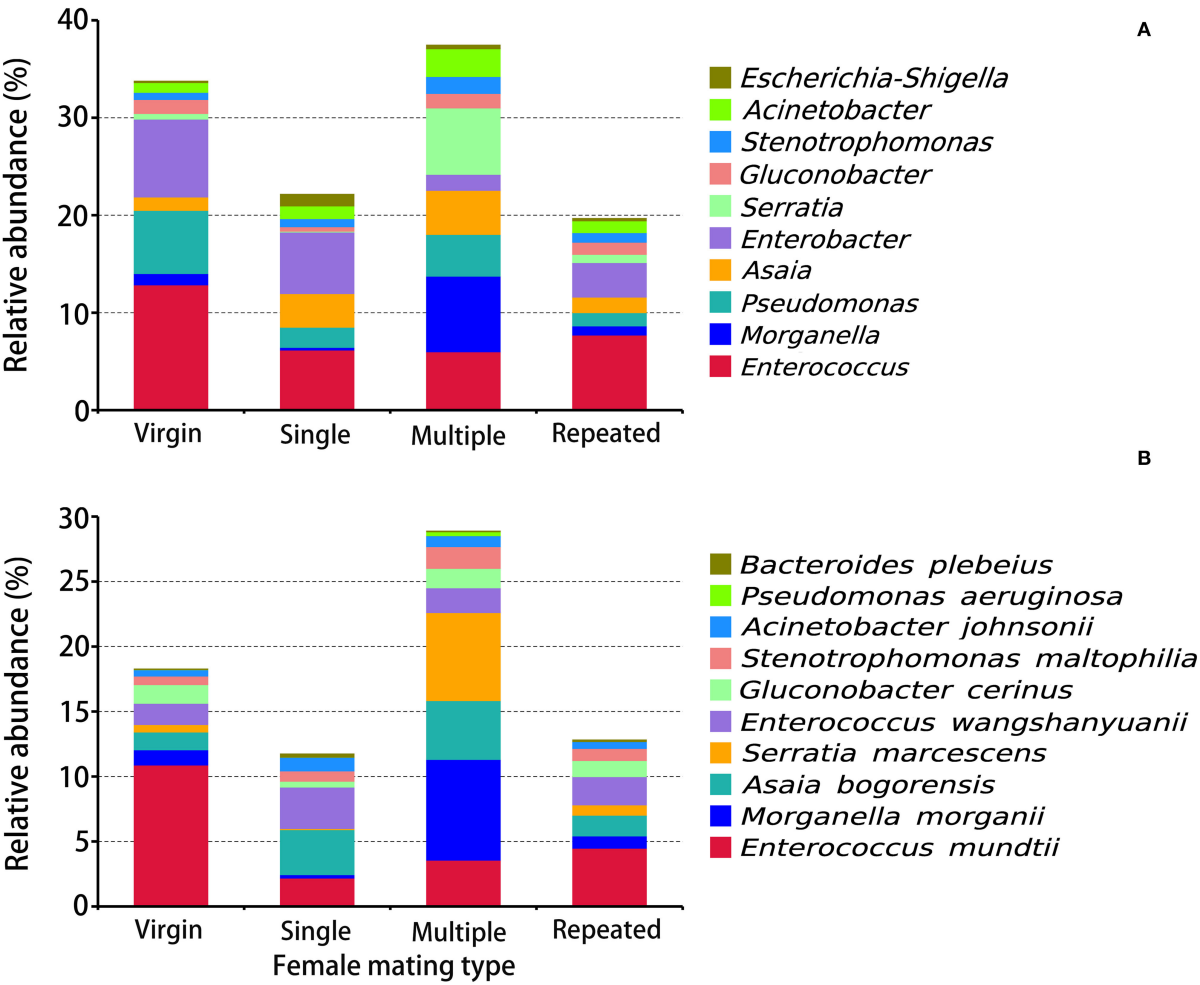
Taxonomy assignment of bacterial OTUs at the genus and species levels in *S. frugiperda* females of different mating types. **(A)** The abundance pattern at the genus level (top 10). **(B)** The abundance pattern at the species level (top 10).

The obtained 2005 OTUs (Supplementary Table 2, Supplementary Figure 2) were classified into 33 phyla (Supplementary Table 5, Figure 3), 81 classes (Supplementary Table 6), 203 orders (Supplementary Table 7), 286 families (Supplementary Table 8), 447 genera (Supplementary Table 9, Figure 4A), and 194 species (Supplementary Table 10, Figure 4B). Within the obtained 2005 OTUs, 605 of them were shared (common OTUs) by females of different mating types (Supplementary Figure 2). The abundance pattern at the phylum level showed that Proteobacteria are the predominant bacterial phylum in *S. frugiperda*, with Multiple showing the highest percentage (90.94%), followed by Repeated with 88.99%, then Single with 86.38%, and Virgin showing the lowest percentage (82.14%) (Figure 3). The second and third dominant Phyla are Firmicutes and Bacteroidota, and both of them showed the highest abundance in virgin females (Figure 3).

OTUs on the genus level (Figure 4A) indicated that *Enterococcus* (12.88%), *Enterobacter* (7.99%), and *Pseudomonas* (6.48%) are the first three dominant genera in Virgin, while in Single are *Enterobacter* (6.30%), *Enterococcus* (6.20%), and *Asaia* (3.47%), in Multiple are *Morganella* (7.76%), *Serratia* (6.82%), and *Enterococcus* (6.01%), and in Repeated are *Enterococcus* (7.72%), *Enterobacter* (3.54%), and *Asaia* (1.59%), indicating remarkable difference on bacterial composition in females from different mating types. The prominent difference on the genus level within females from different mating types is that *Morganella* (7.76%) and *Serratia* (6.82%) are the most dominant genera in Multiple, while in the other mating types their abundance is much lower (0.09–1.17%) (Figure 4A).

OTU clustering into the species level (Figure 4B) disclosed that *Enterococcus mundtii* showed a higher abundance in Virgin (10.90%) than in the other mating types (2.19–4.50%), whereby *Morganella morganii* and *Serratia marcescens* showed higher abundance in Multiple (7.76 and 6.67%) than in the other mating types (0.26–1.16 and 0.09–0.80%).

### Functional Prediction Using FAPROTAX

**Figure 5:**
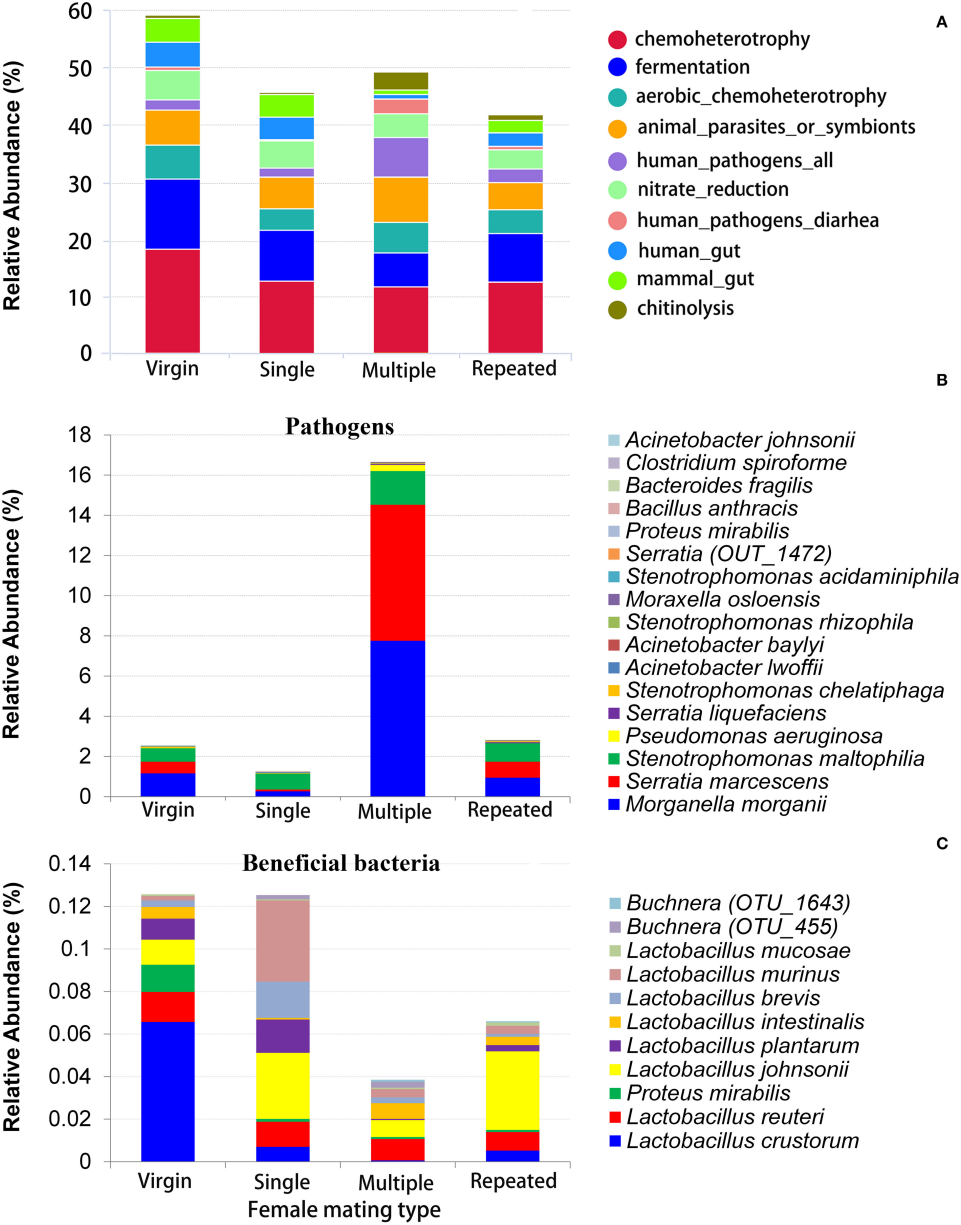
Functional prediction of bacteria in *S. frugiperda* females of different mating types. **(A)** The relative abundance of dominate bacterial functional groups (top 10) distribution predicted by FAPROTAX; **(B)** the relative abundance of possible pathogens; **(C)** the relative abundance of possible beneficial bacteria (bacteria that are beneficial for their hosts).

FAPROTAX was used to evaluate the potential functional differences between different mating types. The predominant function is chemoheterotrophy (responsible for an average 11.33–17.92% of the total annotated functions) in all mating types, followed by fermentation (6.08–12.17%), animal parasites or symbionts (4.66% to 7.76%), and aerobic chemoheterotrophy (3.66–5.94%), and Virgin showed relative higher abundant in all of these functions (Figure 5A, Supplementary Figure 3). The most variable function predicted by FAPROTAX is human pathogens (Figure 5A, Supplementary Figure 3), where Multiple showed the highest abundance on this function (6.94%), followed by Repeated (2.39%), and then Virgin (1.82%), and Single showed the lowest abundance on this function (1.53%).

### Pathogen and Beneficial Bacteria Profiles

Based on the above taxonomy assignment and functional prediction, the possible pathogens and beneficial bacteria were further evaluated based on evidence from published studies. A total of 17 animal pathogens were found, with three of them being insect pathogens and others being pathogens of human and other animal taxa (Table 1). Twelve beneficial bacterial species were found, including eight species from *Lactobacillus*, two species from *Buchnera*, one species from *Proteus*, and one species from *Enterococcus* (Table 1).

**Table 1:** Possible pathogens and beneficial bacteria^*^ profiles in *S. frugiperda*.

**Functional group**	**Taxonomy**			**Functional annotation**	**Reference**
	**Phylum**	**Order**	**Species**		
Pathogen	Proteobacteria	Enterobacterales	*Morganella morganii*	Insect pathogen	Salas et al., 2017
	Proteobacteria	Enterobacterales	*Serratia marcescens*	Insect pathogen	Maciel-Vergara et al., 2018; Voirol et al., 2018
	Proteobacteria	Pseudomonadales	*Pseudomonas aeruginosa*	Insect pathogen	Maciel-Vergara et al., 2018
	Proteobacteria	Enterobacterales	*Serratia liquefaciens*	Animal pathogen	Momose et al., 2018; Voirol et al., 2018
	Proteobacteria	Enterobacterales	*Serratia* spp. (OTU_1472)	Animal pathogen	Voirol et al., 2018; Au et al., 2021
	Proteobacteria	Enterobacterales	*Proteus mirabilis*	Human pathogen	Armbruster et al., 2018; Yuan et al., 2021
	Proteobacteria	Xanthomonadales	*Stenotrophomonas maltophilia*	Human pathogen	Adegoke et al., 2017
	Proteobacteria	Xanthomonadales	*Stenotrophomonas chelatiphaga*	Potential human pathogen	Patil et al., 2021
	Proteobacteria	Xanthomonadales	*Stenotrophomonas rhizophila*	Potential human pathogen	
	Proteobacteria	Xanthomonadales	*Stenotrophomonas acidaminiphila*	Potential human pathogen	Huang et al., 2018
	Proteobacteria	Pseudomonadales	*Acinetobacter lwoffii*	Human and fish pathogen	Peleg et al., 2008; Kozińska et al., 2014
	Proteobacteria	Pseudomonadales	*Acinetobacter johnsonii*	Human and fish pathogen	
	Proteobacteria	Pseudomonadales	*Acinetobacter baylyi*	Human pathogen	Chen et al., 2008
	Proteobacteria	Pseudomonadales	*Moraxella osloensis*	Human pathogen	Maruyama et al., 2018
	Firmicutes	Bacillales	*Bacillus anthracis*	Human pathogen	Passalacqua and Bergman, 2006
	Bacteroidota	Bacteroidales	*Bacteroides fragilis*	Human pathogen	Martin and Aziz, 2009
	Firmicutes	Erysipelotrichales	*Clostridium spiroforme*	Human and rabbit pathogen	Agnoletti et al., 2008; Carter et al., 2014
Beneficial bacteria	Firmicutes	Lactobacillales	*Enterococcus mundtii*	Against entomopathogens	Shao et al., 2017; Voirol et al., 2018
	Firmicutes	Lactobacillales	*Lactobacillus crustorum*	Healthy vaginal microbiome	Schoenmakers et al., 2019; Rowe et al., 2021
	Firmicutes	Lactobacillales	*Lactobacillus reuteri*	Healthy vaginal microbiome	
	Firmicutes	Lactobacillales	*Lactobacillus johnsonii*	Healthy vaginal microbiome	
	Firmicutes	Lactobacillales	*Lactobacillus plantarum*	Healthy vaginal microbiome	
	Firmicutes	Lactobacillales	*Lactobacillus intestinalis*	Healthy vaginal microbiome	
	Firmicutes	Lactobacillales	*Lactobacillus brevis*	Healthy vaginal microbiome	
	Firmicutes	Lactobacillales	*Lactobacillus murinus*	Healthy vaginal microbiome	
	Firmicutes	Lactobacillales	*Lactobacillus mucosae*	Healthy vaginal microbiome	
	Proteobacteria	Enterobacterales	*Proteus mirabilis*	Colonization resistance	Dillon and Dillon, 2004
	Proteobacteria	Enterobacterales	*Buchnera* spp. (OTU_455)	Nutritional contributions	
	Proteobacteria	Enterobacterales	*Buchnera* spp. (OTU_1643)	Nutritional contributions	

* *Bacteria that are beneficial for their hosts*.

Statistics indicated that the Multiple has the most abundance on pathogens (16.65%), which is more than five times than that of the other mating types (1.26–2.81%) (Figure 5B). On the contrary, Multiple showed the lowest abundance on beneficial bacteria (see Figure 4B for *Enterococcus mundtii* and Figure 5C for other beneficial bacteria).

**Figure 6:**
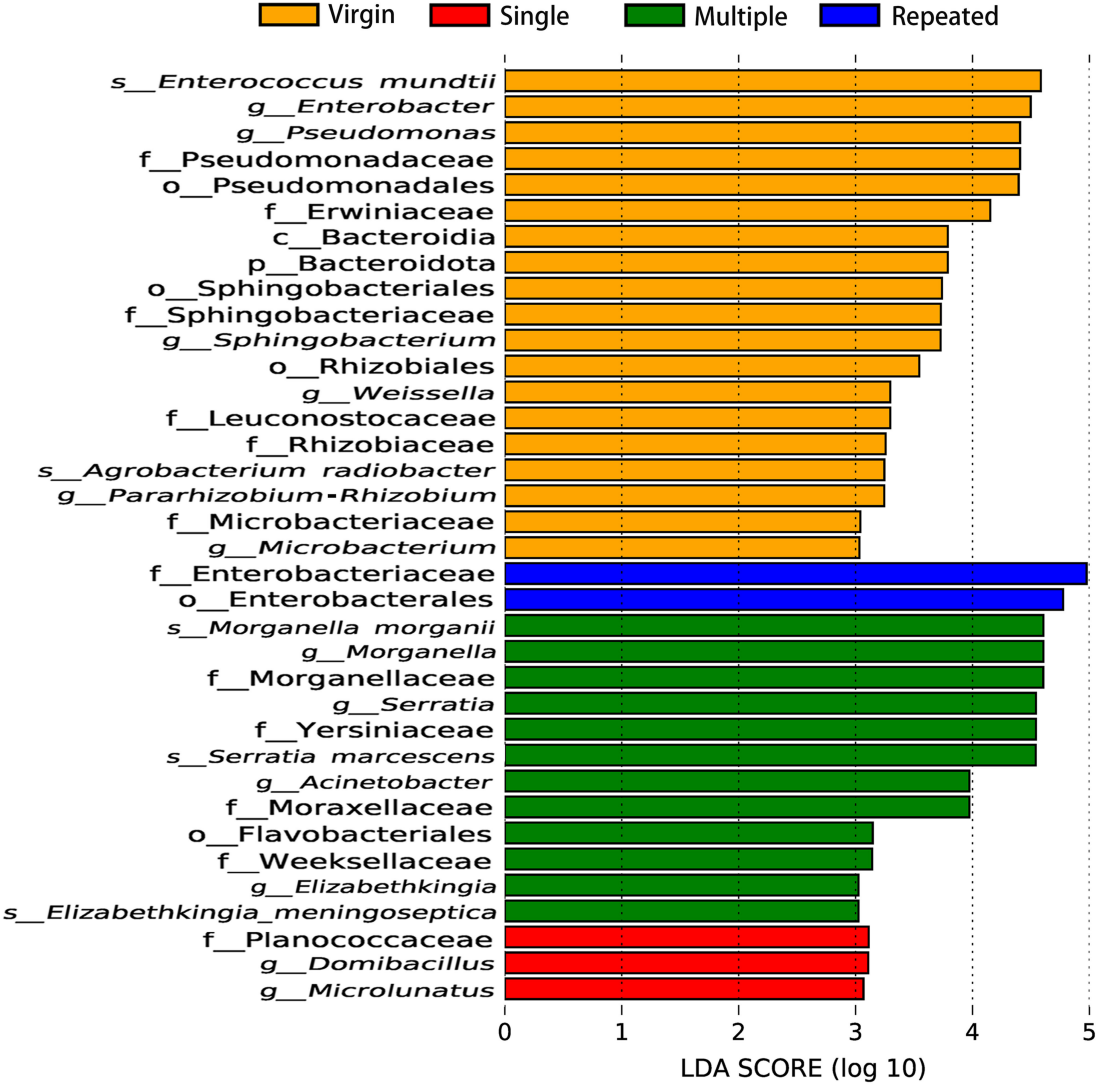
Important biomarkers of bacterial communities in *S. frugiperda* females of different mating types revealed by linear discriminant analysis (LDA).

Linear discriminant analysis (LDA) also showed remarkable differences on the number and taxon of microbial biomarkers between different mating types. More microbiomes were detected as important biomarkers in Virgin and Multiple than in Single and Repeated, with one beneficial bacteria (*Enterococcus mundtii*) showing in the Virgin while two pathogens (*Morganella morganii* and *Serratia marcescens*) in the Multiple (Figure 6). The OTUs represented in each population also were illustrated by phylogenetic levels from phylum to genus in the cladogram (Supplementary Figure 4).

## Discussion

The microbiomes associated with animal reproduction are opportunistic and/or sexually transmittable, which may affect host immune defense and future colonization of other microorganisms. Only a few studies have studied the microbiomes in the reproductive organs and guts in insects (Wang et al., 2014; Otti et al., 2017; Bellinvia et al., 2020a,b) and tested the effect of mating on the composition of microbiomes (Bellinvia et al., 2020a,b). In this study, we considered the symbiotic microbiomes in the abdomen as a whole in a community context and tested the diversity and abundance of microbiomes under different mating conditions (Figure 1).

Beta-diversity analysis based on Bray–Curtis distance showed significant variances in the composition of OTUs in females with different mating statuses (Figures 2D–F), suggesting that mating significantly affected the composition of microbiomes in the abdomen of *S. frugiperda* females. The composition of genital microbiomes of females should be more affected by the sexual transmission of bacterial strains, via male genitalia and the ejaculate, and by bacteria transferred from the cuticle to the reproductive duct during and after mating (Lange et al., 2013; Reinhardt et al., 2015). Moreover, these invading bacteria may enter the hemolymph and other organs via reproductive duct and copulatory wounds (Lange et al., 2013; Reinhardt et al., 2015; Bellinvia et al., 2020a). Previous studies in a number of insect species have found pronounced differences in the microbiomes of reproductive organs between females and males (Wang et al., 2014; Otti et al., 2017; Bellinvia et al., 2020a,b). Bellinvia et al. (2020a,b) further demonstrated that bacteria are sexually transmitted in the common bedbug, *C. lectularius*. However, results in *C. lectularius* show that mating did not cause significant effect on alpha diversity of bacteria in the reproductive organs (Bellinvia et al., 2020b). In this study, alpha diversity indicated that the OTU diversity was significantly different in *S. frugiperda* females with different mating statuses, and mated females showed significant lower diversity than virgin females (Figure 2A) and in more details, virgin females have the highest diversity, followed by one-time mated females and females mated with multiple males, while females mated repeatedly showed the lowest diversity (Figures 2B,C). On the one hand, mating is likely to promote the diversity of microbes due to sexual transmission (Bellinvia et al., 2020a,b). On the other hand, mating may upregulate female immune responses (Oku et al., 2019), particularly in females with polygamous mating systems (Okada et al., 2017; Oku et al., 2019), which may affect the composition and diversity of microbes inside their bodies. In our study, females mated repeatedly with the same mates showed the lowest diversity (Figures 2B,C), which may be due to lower sexual transmission as only mated with one male but higher immune response from repeated matings.

In this study, the obtained 2005 OTUs were classified into 33 phyla, with Proteobacteria, Firmicutes, and Bacteroidota being the first three dominant phyla (Figure 3). Matings further increased the abundance of Proteobacteria (up to 90%) but decreased the abundance of Firmicutes and Bacteroidota (Figure 3). In the citrus fruit fly, *Bactrocera minax*, 16S rDNA sequencing revealed that Proteobacteria were dominant in gut and reproductive organs (Wang et al., 2014). Other studies have also found an abundance of Proteobacteria in various insect species, such as the sawfly *Cephalcia chuxiongica* (Yu et al., 2021), the desert locust *Schistocerca gregaria* (Dillon et al., 2010), the *Lutzomyia* sand fly (Sant'anna et al., 2012), and two ground beetles (Lundgren et al., 2007). Proteobacteria may play important roles in insects, such as help insects to fix nitrogen and preventing the establishment or proliferation of pathogenic bacteria (Dillon and Dillon, 2004; Dixon and Kahn, 2004; Behar et al., 2005). OTUs clustering into the genus (Figure 4A) also revealed significant differences in females from different mating types. For example, *Morganella* and *Serratia* are the most dominant genus in multiple mated females, while in other mating types their abundances are much lower. Further, *Enterococcus mundtii* showed a higher abundance in virgin females than in females from the other mating types, while *Morganella morganii* and *Serratia marcescens* showed higher abundances in multiple mated females than in females from the other mating types (Figure 4B). Species from *Enterococcus* may play a protective role against insect pathogens in the lepidopteran intestine (Voirol et al., 2018). For example, *E. faecalis* found in the gypsy moth can acidify its local environment to facilitate it colonize alkaline niches, which may help to host against pathogenic toxins that are activated in alkaline conditions, such as those from *Bacillus thuringiensis* (Broderick et al., 2004). *E. mundtii* is a highly abundant bacterium in the gut of *S. littoralis*, which is known to produce antimicrobial compounds that act against gram-positive pathogens (such as *Listeria*), but not against resident gut bacteria (Shao et al., 2017; Voirol et al., 2018). *M. morganii* is a lethal pathogen of Mexican fruit fly larvae (Salas et al., 2017).

Some common gut bacterial inhabitants can be beneficial or detrimental depending on the community composition of the gut. *Serratia* spp. is one of such bacteria, which is a genus known to be pathogenic in many animals including insects (Chadwick et al., 1990; Ishii et al., 2014; Momose et al., 2018; Voirol et al., 2018). For example, the SM1 strain of *S. marcescens* was highly toxic to the black-winged termite, *Odontotermes formosanus*, probably through the production of insecticidal protease and metalloproteinases (Fu et al., 2021). *Serratia* spp. are commonly reported in lepidopterans, while their roles are unclear in terms of detrimental or beneficial effects (Broderick et al., 2004). Future studies should evaluate whether such bacteria exert no negative effects or provide benefits on insect health depending on the composition of the entire insect microbial community and whether they switch from a symbiont to a systemic pathogen when the environmental condition is altered, such as postmating.

Functional prediction by FAPROTAX also disclosed a most variable on the function of human pathogens all in *S. frugiperda* females from different mating types, where females mated with multiple males showed higher abundance on this function than females from other mating types (Figure 5A, Supplementary Figure 3).

Due to the turnover on beneficial and detrimental microbiomes between virgin and multiple mated females found by taxonomy assignment and functional prediction by FAPROTAX, the possible pathogens and beneficial bacteria were further determined based on evidence from previous studies. The FAPROTAX and evidence-based double checking ensure the credibility of the functional evaluation of bacteria. Three insect pathogenic species and 14 pathogenic species from human and other animal taxa were verified by previous published studies (Table 1). Twelve beneficial bacterial species from four genera, *Lactobacillus, Buchnera, Proteus*, and *Enterococcus*, were also verified by published studies (Table 1). *Buchnera* has been implicated in various nutritional and non-nutritional functions in insects, such as provisioning of essential amino acids and nitrogen recycling (Douglas, 1998; Dillon and Dillon, 2004). Studies in human found that the *Lactobacillus* spp. are beneficial vaginal microbiomes, which may function in barring against pathogen adhesion, production of lactic acid against the colonization of pathogens, and stimulating host defense responses (Ravel et al., 2011; Chen et al., 2017; Tachedjian et al., 2017; Younes et al., 2018). The function of *Lactobacillus* spp. in insects is still unclear and thus warrants further studies.

Statistics showed that females mated with multiple males have the highest abundance on pathogens (more than five times than that of females from the other mating types) (Figure 5B) while they have the lowest abundance on beneficial bacteria (Figures 4B, 5C) in comparison with that of females from the other mating types. Moreover, LDA demonstrates that the *Enterococcus mundtii* (a beneficial bacterium) is one of the important biomarkers in virgin females, whereas *Morganella morganii* and *Serratia marcescens* (pathogens) are two of the important biomarkers in multiple mated females (Figure 6), further supporting the hypothesis that promiscuity turned over the abundance of beneficial and detrimental microbiomes in *S. frugiperda* females.

However, whether and how these possible pathogens and beneficial bacteria affect the health and reproduction of *S. frugiperda* still needs to be validated by further studies. Moreover, future studies by comparing microbiomes of reproductive organs and non-reproductive organs of both sexes under different mating conditions are expected to achieve deeper insight in this field.

## Data Availability Statement

The datasets presented in this study can be found in online repositories. The names of the repository/repositories and accession number(s) can be found in the article/Supplementary Material.

## Author Contributions

L-YZ and HYu collected the insects. L-YZ, D-YF, and JX designed the study. L-YZ, HYu, and D-YF dissected the insects and extracted DNA. L-YZ, SY, HYe, and JX analyzed the data. L-YZ, JX, and HYe wrote the paper. All authors read and approved the final manuscript.

## Funding

This study was jointly supported by projects from Science and Technology Planning Project in Key Areas of Yunnan Province (202001BB050002), Basic Research Projects of Yunnan Province (202201AS070025), Joint Special Project of Yunnan Province for Agricultural Basic Research (2018FG001-002), and National Natural Science Foundation Program of P.R. China (31760635, 31560606).

## Conflict of Interest

The authors declare that the research was conducted in the absence of any commercial or financial relationships that could be construed as a potential conflict of interest.

## Publisher's Note

All claims expressed in this article are solely those of the authors and do not necessarily represent those of their affiliated organizations, or those of the publisher, the editors and the reviewers. Any product that may be evaluated in this article, or claim that may be made by its manufacturer, is not guaranteed or endorsed by the publisher.

## Supplementary Material

Supplementary Figure 1 Rarefaction curves represent observed OTUs in different samples.

Supplementary Figure 2 The Venn **(A)** and petals **(B)** diagrams of OTUs from different mating types.

Supplementary Figure 3 The heat map of bacterial functions (top 35) in the FAPROTAX database for *S. frugiperda* females. The samples are grouped according to the similarity of each other. Different colors indicate the relative abundance of groups in the individual samples, wherein red represents the function with higher abundance, and blue represents the function with lower abundance in the corresponding sample.

Supplementary Figure 4 Cladogram of the LDA results, where rings from the inner to outer indicate phylum, class, order, family, and genus, respectively.
