# Supplementary figures and images for: Mating Leads to a Decline in the Diversity of Symbiotic Microbiomes and Promiscuity Increased Pathogen Abundance in a Moth
Source: Front Microbiol. 2022 May 12;13:878856. doi: 10.3389/fmicb.2022.878856 (PMC9133953; doi:10.3389/fmicb.2022.878856)

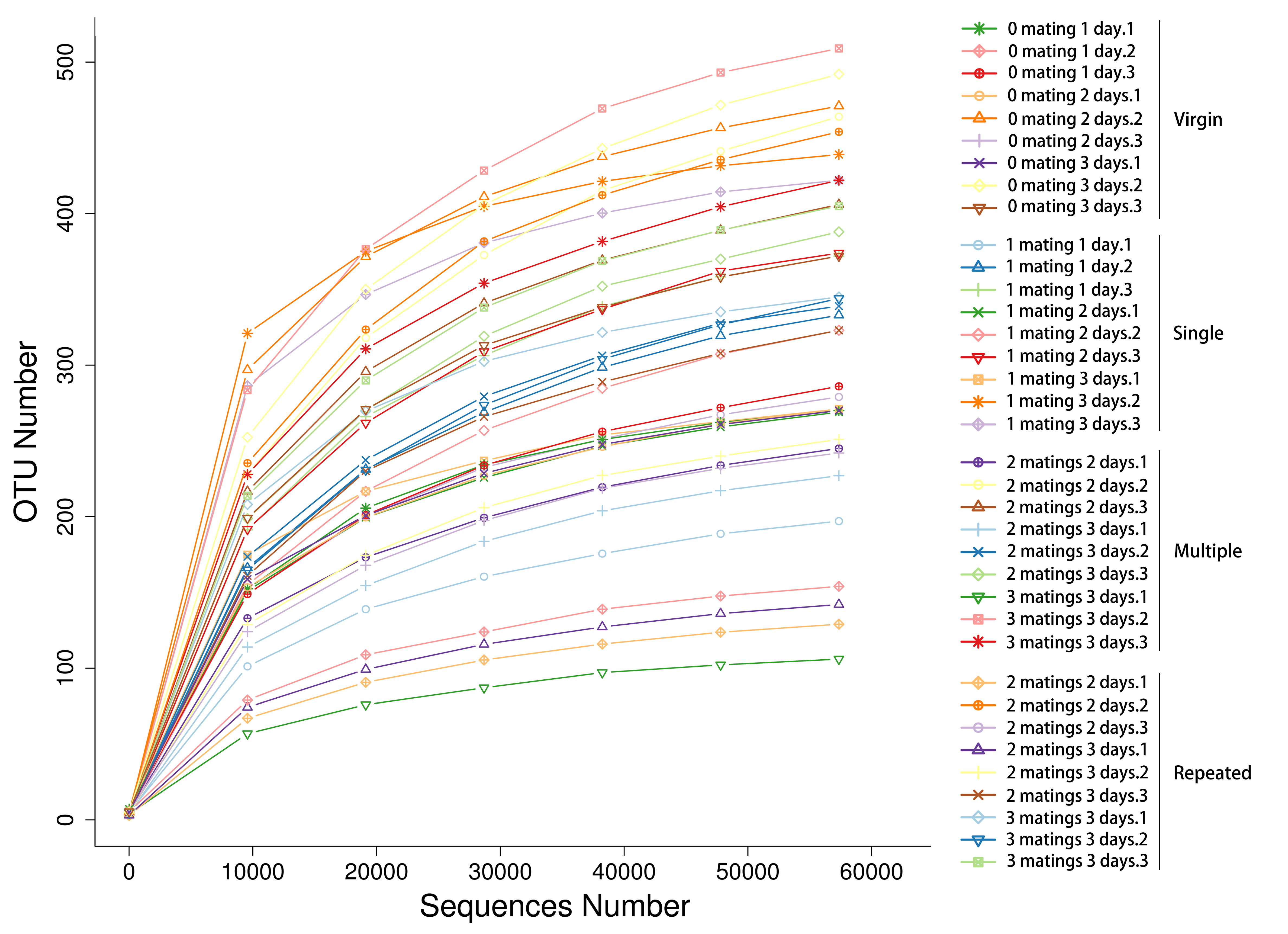

Supplement: Supplementary Figure 1 — Rarefaction curves represent observed OTUs in different samples. [file Image_1.TIFF]

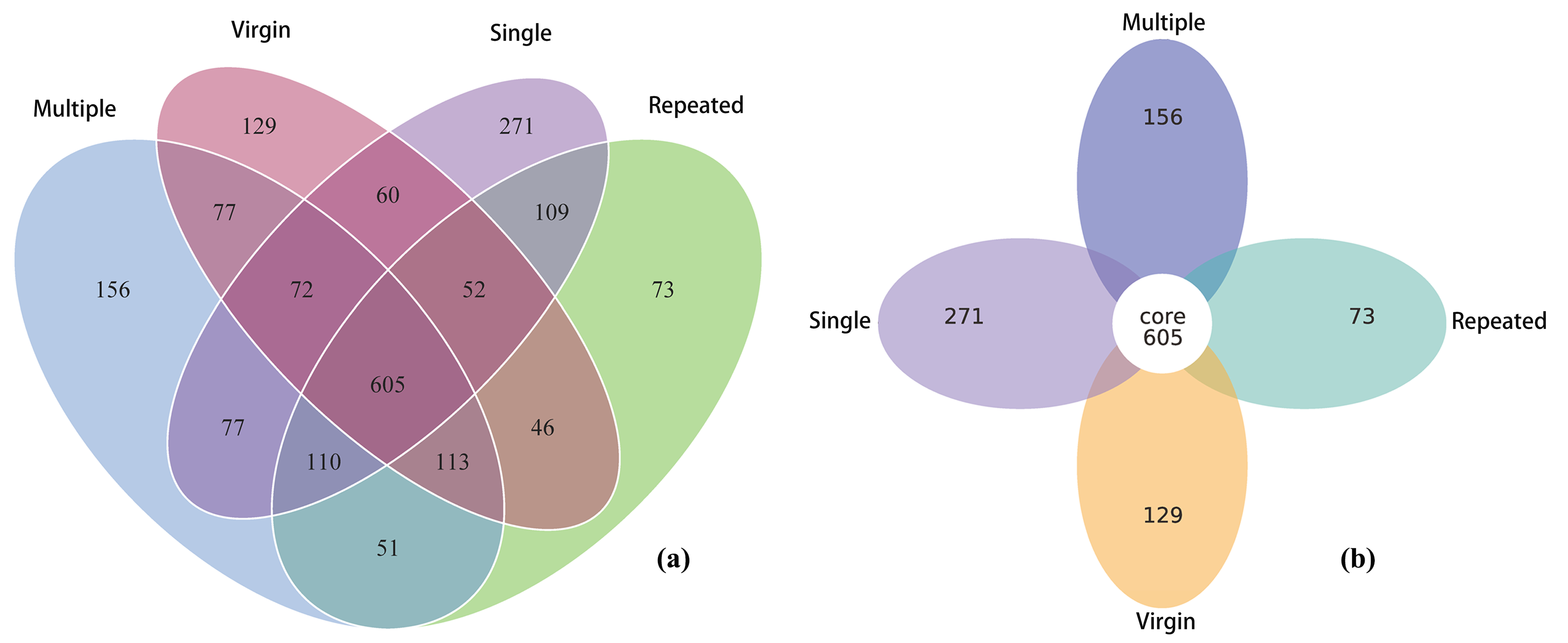

Supplement: Supplementary Figure 2 — The Venn (A) and petals (B) diagrams of OTUs from different mating types. [file Image_2.TIF]

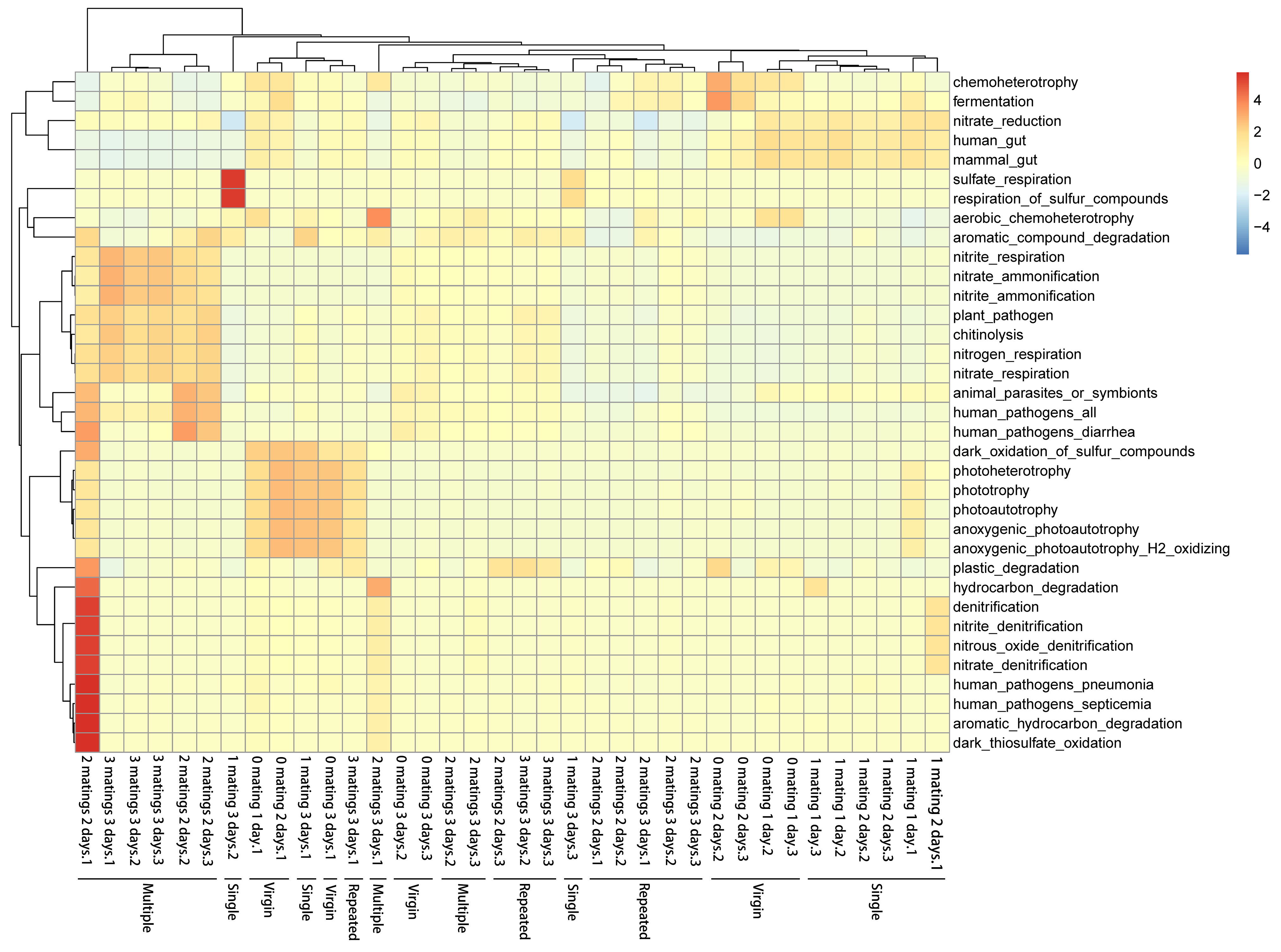

Supplement: Supplementary Figure 3 — The heat map of bacterial functions (top 35) in the FAPROTAX database for S. frugiperda females. The samples are grouped according to the similarity of each other. Different colors indicate the relative abundance of groups in the individual samples, wherein red represents the function with higher abundance, and blue represents the function with lower abundance in the corresponding sample. [file Image_3.TIF]

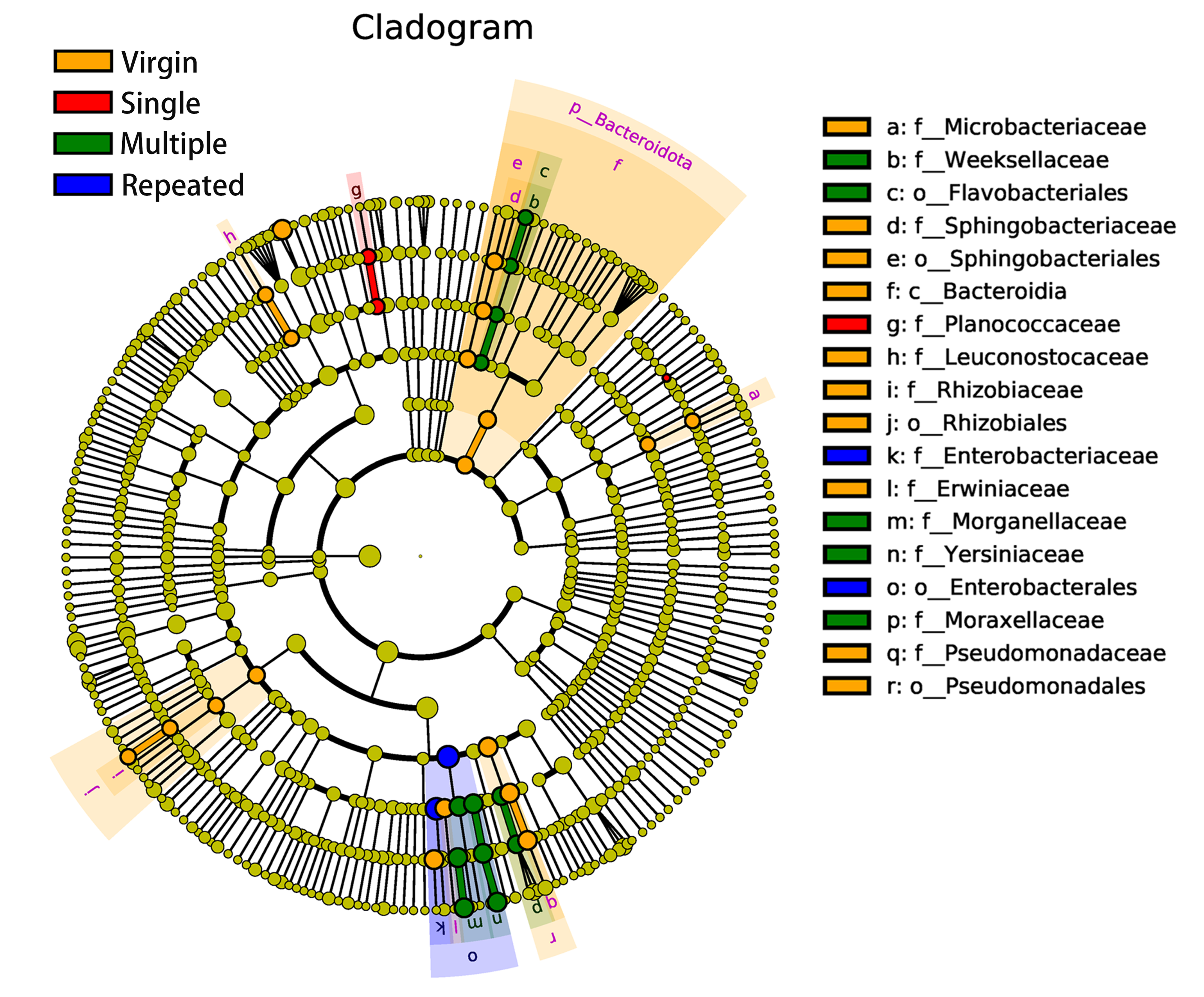

Supplement: Supplementary Figure 4 — Cladogram of the LDA results, where rings from the inner to outer indicate phylum, class, order, family, and genus, respectively. [file Image_4.TIF]
